# The Effect of Aligned and Random Electrospun Fibrous Scaffolds on Rat Mesenchymal Stem Cell Proliferation

**Published:** 2012-06-13

**Authors:** Hoda Jahani, Saeid Kaviani, Majid Hassanpour-Ezatti, Masoud Soleimani, Zeinab Kaviani, Zahra Zonoubi

**Affiliations:** 1. Department of Physiology, Faculty of Basic Sciences, Shahed University, Tehran, Iran; 2. Department of Hematology, Faculty of Medical Sciences, Tarbiat Modares University, Tehran, Iran; 3. Department of Medical Sciences, Faculty of Karaj Medical Sciences, Karaj, Iran; 4. Department of Obstetrics and Gynecology, Mahdiyeh Hospital, Shahid Beheshti University, Tehran, Iran

**Keywords:** Tissue Engineering, PCL, Surface Modification, Mesenchymal Stem Cells

## Abstract

**Objective:**

The development of combining mesenchymal stem cells (MSCs) with surface modified three-dimensional (3D) biomaterial scaffold provides a desirable alternative for replacement of damaged and diseased tissue. Nanofibrous scaffolds serve as suitable environment for cell attachment and proliferation due to their similarity to the physical dimension of the natural extracellular matrix. In this study the properties of plasma treated poly-C-caprolactone nanofiber scaffolds (p-PCL) and unaltered PCL scaffolds were compared, and then p-PCL scaffolds were evaluated for MSC culture.

**Materials and Methods:**

Aligned and random PCL nanofibrus scaffolds were fabricated by electrospining and their surface modified with O_2_ plasma treatment to enhance MSC proliferation, adhesion and interaction. Chemical and mechanical characterizations were carried out using scanning electron microscopy (SEM), water contact angle and tensile testing. Cell adhesion and morphology were evaluated using SEM 1 day after culture. Statistical analysis was carried out using one way analysis of variance (ANOVA).

**Results:**

The proliferation of MSCs were evaluated using 3-(4,5-Dimethylthiazol-2-yl)-2,5-Diphenyltetrazolium Bromide(MTT) assay on day 1, 3, and 5 after cell culture. Results showed that the numbers of cells that had grown on PCL nanofibrous scaffolds were significantly higher than those of control surfaces without nanofibers. Furthermore, the proliferation of MSCs on random nanofiber was significantly higher compared to that on aligned nanofiber.

**Conclusion:**

This study showed that while both aligned and random plasma treated PCL nanofibrous scaffold are more suitable substrates for MSC growth than tissue culture plates, random nanofiber best supported the proliferation of MSCs.

## Introduction

Recently, tissue engineering and three- dimensional (3-D) scaffolds have been developed to address the extreme shortage of tissues and organs for transplantation and repair. Tissue engineering has provided new medical therapy as an alternative to conventional transplantation methods using polymeric biomaterials with precursor cells. Fundamentally, tissue engineering involves the *in vitro* seeding of cells onto scaffolds in order to promote cell adhesion, proliferation and differentiation due to similarity of the environment to the extracellular matrix (ECM) (1, 2). *in vitro* system scaffolds can play an effective role in controlling cell behavior in the same way as the ECM in a living system. Among the various types of scaffolds available, scaffolds based on nanofibers which mimic the structure of the ECM offer great advantages. The development of biodegradable polymeric scaffolds with surface properties that control interactions between the material and the biological environment is of great interest in biomedical applications (2, 3).

Synthetic polymers represent the largest class of bio- material. A wide variety of synthetic polymers have been used to form nanofiber because their mechanical properties and degradation rates can be tailored to suit a wide range of special applications (4- 6). PCL is an aliphatic, biodegradable and non-toxic polyester that has good mechanical properties. However, its low hydrophilicity, together with a lack of functional groups, often results in low cell adhesion and proliferation on these scaffolds (7, 8). Different types of physicochemical and post-processing surface modification techniques have been attempted to solve the problem of cell adhesion and proliferation on hydrophobic surfaces (8). In addition, properties that can be further altered to make hydrophobic surfaces more effective as biomaterial have been investigated. Plasma treatment is a useful method for altering the hydrophobicity properties of PCL by introducing desired functional groups onto the surface of the scaffold (9, 10). The extent of cell attachment and growth on polymer surfaces are important factors that directly influence the capacity of cells to proliferate and differentiate on these polymeric scaffolds (11, 12).

 MSCs are non-hematopoietic progenitor cells with the potential to differentiate into cells of the mesoderm lineage. MSCs are an easily accessible cell source for tissue engineering applications (13, 14). Interest in nano engineering of MSCs is mainly due to their potential for transplantation, regeneration and treatment of degenerative and autoimmune diseases (7). Reports are available on the proliferation of other cells on PCL nanofibrous scaffold (3, 8). However, very few studies have focused on the proliferation of MSCs on electrospun nanofibrous substrates (7, 15). For this reason we studied the efficacy of PCL nanofiber scaffolds using MSCs to assess their potential as substrates for tissue engineering applications. Our study aimed to produce a biocompatible 3-D nanofibrous scaffold supporting the proliferation of MSCs that could be used as a tissue engineered cell-scaffold construct for transplantation. Based on the above rationale, aligned and random PCL scaffolds were made by electrospinning and their properties; such as hydrophilisity, porosity, tensile strength and ability to support the growth, proliferation and attachment of MSCs were evaluated compared to a 2-D culture system.

## Materials and Methods

### Materials

Poly-C-caprolactone with a molecular weight of 80 000, chloroform, dimethyl formaldehyde (DMF) were purchased from Sigma-Aldrich (St Louis, MO, USA). Rat MSCs were obtained from the Stem Cell Technology Research Center (Tehran, Iran). Dulbecco’s Modified Eagle’s Medium (DMEM) was obtained from Sigma; fetal bovine serum (FBS), antibiotics and trypsin-EDTA were purchased from GIBCO Invitrogen (Carlsbad, CA, USA).

### Electrospinning of nanofibers

PCL (8 wt%) was dissolved in chloroform/DMF (9:1) for the electrospinning process. Polymer solution was loaded into a 5 ml plastic syringe with a 21 G needle and fed to the needle tip using a syringe pump at a flow rate of 0.5 ml per hour. A positive voltage of 25 kV was applied to the needle using a high voltage power supply. For collecting aligned nanofibers, a rotating disk with a linear rate set to 1000 rpm was used, whereas a rotating disk with a linear rate set to 100 rpm was used for collecting random nanofibers. The collector was placed at a distance of 23 cm from the needle tip. On applying a high voltage the polymer solution formed a Taylor cone at the needle tip and a positively charged jet was sprayed on the collector. Nanofibers collected on aluminum plates were dried overnight under vacuum before being used for experiments.

### Surface modification of electrospun nanofibers

O_2_ plasma treatment of electrospun PCL nanofibrous scaffolds was carried out using a Diener electronic plasma cleaner (Germany). Nanofibers were placed in the chamber of the plasma cleaner and plasma discharge was applied for 3 minuets with radio frequency power set as 30 W under vacuum mode.

### Physicochemical and mechanical characterization of nanofibers

Electrospun nanofibrous scaffolds were sputter coated with gold (Polaron SC 7620 sputter coater) and examined under a field emission scanning electron microscope (LEO 1455VP, England) at an accelerating voltage of 10 kV.

The water contact angle for PCL nanofibrous scaffolds before and after plasma treatment was measured using a Contact Angle measuring System G10 (KRűSS, Korea), mounted with a CCD camera. Scaffolds were placed on the sample stage and a drop of distilled water was dropped to the surface for contact angle measurement.

Mechanical testing was carried out using a tensile tester (SANTAM Stress machine STM20, Iran) at a speed rate of 50 mm min^-1^. Rectangular specimens were cut from the as-spun membranes with dimensions 60×10 mm^2^ used for mechanical testing studies. The ends of the rectangular specimens were mounted vertically on mechanical gripping units of the tensile tester. The results were plotted to obtain the stress–strain curve for scaffolds.

The bulk density of PCL is 1.146 g/ cm^3^. The thicknesses of PCL and plasma treated PCL (p-PCL) random and aligned nanofibrous membranes were measured by micrometer, and apparent densities and porosities were calculated using the following equations:

$$\mathrm{Porosity(\%)}=\left(1-\frac{\mathrm{Apparent Density}\left(\frac{g}{{\mathrm{cm}}^{3}}\right)}{\mathrm{Bulk density of membranes}\left(\frac{g}{{\mathrm{cm}}^{3}}\right)}\right)\times 100\%$$

$$\mathrm{Apparent Density}\left(\frac{g}{{\mathrm{cm}}^{3}}\right)=\frac{\mathrm{Mass of nanofiber membrane (g)}}{\mathrm{membrane thickness (cm)}\times \mathrm{area}({\mathrm{cm}}^{2})}$$

### In vitro culture of mesenchymal stem cells

Mesenchymal stem cells obtained from the Stem Cell Technology Research Center (Tehran, Iran) were maintained in Dulbecco’s modified Eagle’s medium (DMEM) supplemented with 10% fetal bovine serum (FBS) and 1% penicillin/streptomycin/amphotericin-B (Invitrogen Corp, USA). Cells were maintained in a humidified CO_2_ incubator at 37 ◦C until confluency and fed with fresh medium every 3 days. Before seeding, cells were detached from the cell culture flask with trypsin-EDTA and cells were counted using a Neubauer lam. The second passage culture was used for proliferation and SEM experiments.

### MTT assay for mesenchymal stem cell proliferation

Rat mesenchymal stem cells were seeded (10 000 cells cm−2) on 24-well Tissue culture plates (TCP) for comparisons of control, random and aligned p-PCL nanofibrous scaffolds. Cell proliferation was monitored after 1, 3, and 5 days by MTT assay [3-(4, 5-dimethylthiazol-2-yl)-2, 5-diphenyltetrazolium bromide, a yellow tetrazole]. The number of cells on different scaffolds obtained by MTT colorimetric assay determines the MSCs adhesion and proliferation capacity on different nanofibrous scaffolds. Metabolically active cells react with tetrazolium salt in the MTT reagent to produce a soluble formazan dye, measured at 540 nm. After designated time periods, the cell constructs were rinsed with phosphate buffered saline (PBS) and incubated with 10% MTT reagent in DMEM for 3 h. The color developed dye was further pipetted out in 96-well plates and absorbance was read using an Eppendorf Bio Photometer (Germany).

### Morphology of mesenchymal cells

Morphological studies of the *in vitro* cultured Mesenchymal stem cells on p-PCL aligned and random nanofibrous scaffolds were performed. After 1 day of cell seeding, the cell-cultured scaffolds were processed for SEM studies. The scaffolds were rinsed twice with PBS and fixed in 2.5% glutaraldehyde for 3 hours. Thereafter, the scaffolds were dehydrated with upgrading concentrations of ethanol (60%, 70%, 80%, 90% and 100%) for 15 minutes each. Finally the scaffolds were sputter coated with gold and then observed under SEM.

#### Statistical analysis

Data were obtained at least in triplicate (n=3) averaged and expressed as mean ± standard deviation (SD). Statistical analysis was carried out using one way analysis of variance (ANOVA). A value of p ≤ 0.05 was considered statistically significant.

## Results

### Chemical and mechanical properties of electrospun nanofibrous scaffolds

**Fig 1:**
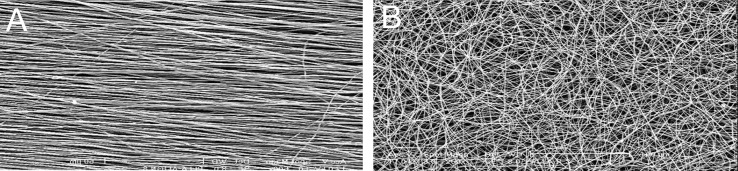
SEM micrograph of nanofiber:(A) aligned PCL nanofiber; (B) random PCL nanofiber. Scale bar: 100 µm

SEM micrographs of electrospun nanofibrous scaffolds revealed porous, nanoscaled fibrous structures formed under controlled parameters. Aligned PCL nanofibers showed a consistent thickness with fiber diameters of 400-1500 nm (Fig 1A). Randomly oriented PCL nanofibrous scaffolds produced uniform, bead free nanofibers and with fiber diameters in the range of 400-1500 nm (Fig 1B). SEM images didn’t show any changes in the surface morphology of PCL nanofiber after plasma treatment.

Contact angle studies of PCL and p-PCL nanofiber scaffolds revealed the surface hydrophilic properties. The contact angles obtained for aligned and random PCL scaffolds were about 130 and 134 respectively, which imply that these scaffolds were highly hydrophobic and nonabsorbent to water. The p-PCL nanofibrous scaffolds were extremely hydrophilic with their water contact angle being less than 80˚ showing 100% wettability by the water droplet and signifying the presence of hydrophilic groups on the surface of the scaffold. A comparison between the fiber properties of PCL and p-PCL random nanofibrous scaffolds is illustrated in table 1.

**Table 1:** Comparison of nanofiber properties of PCL and P-PCL aligned and random nanofibers


Properties	Aligned PCL	Aligned p-PCL	Random PCL	Random p-PCL

Porosity	99	99	99	99
Contact angle (Deg)	130	< 80	134	< 80
Wettability	Highly hydrophobic	Highly hydrophilic	Highly hydrophobic	Highly hydrophilic


**Fig 2:**
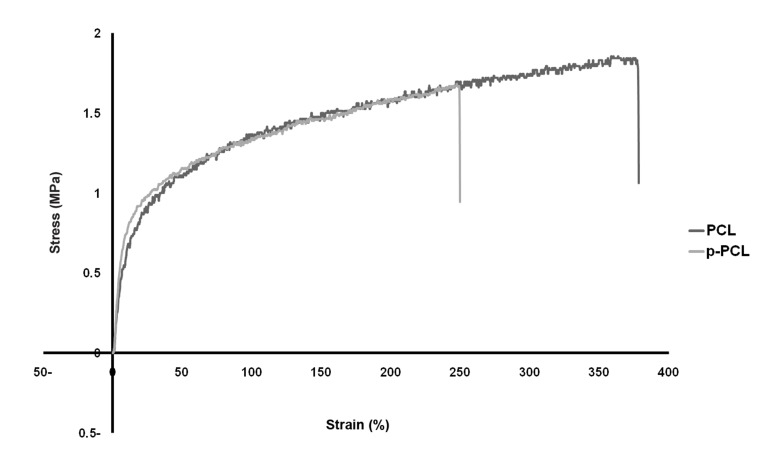
Stress-strain of PCL and p-PCL random nanofiber.

**Fig 3:**
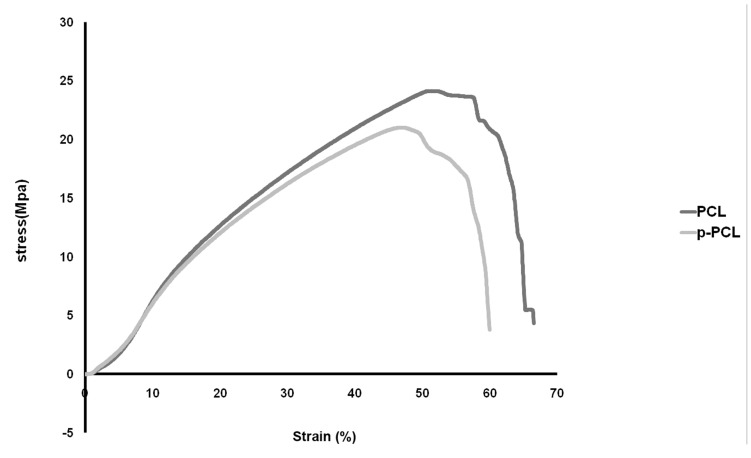
Stress-strain curve of PCL and p-PCL aligned nanofiber

PCL and p-PCL nanofibers showed a typical non-linear stress–strain curve as illustrated in figures 2, 3. The random and aligned p-PCL nanofibrous scaffold showed a reduction in mechanical strength. It is suggested that the hydrophilicity of p-PCL scaffolds decreased their mechanical strength. The elongation of p-PCL nanofibers was lower than that of PCL nanofibers. Table 2 documents the mechanical properties of PCL and p-PCL random and aligned nanofibrous scaffolds with respect to tensile stress and strain.

**Table 2:** Tensile properties of PCL nanofibers


Nanofiber scaffold	Tensile stress	Tensile strain

Random PCL	1.85	363.79
Random p-PCL	1.68	247.39
Aligned PCL	24.11	51.37
Aligned p-PCL	20.97	47.2


### Cell proliferation

**Fig 4:**
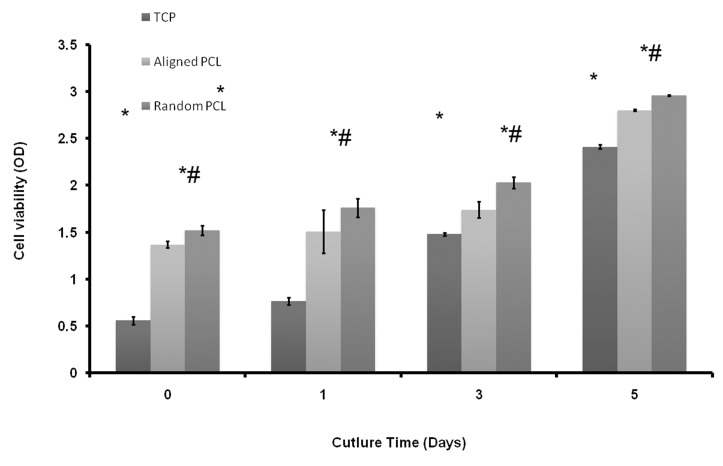
MTT assay for Mesenchymal cell proliferation on p-PCL aligned and random nanofiber scaffold and TCP. Bar represents mean ± standard deviation. * Indicates significant level of proliferation compared to TCP at ≤ 0.05; # indicates significant level of proliferation on random nanofiber compared to aligned scaffold at ≤ 0.05. OD, optic density.

The proliferation of Mesenchymal stem cells on p-PCL nanofibrous scaffolds was significantly (p ≤ 0.05) higher than the cell proliferation on TCP. The results obtained from MTT assay indicated that p-PCL nanofibrous scaffolds were more suitable substrates than TCPs in relation to cell attachment and proliferation. However, the proliferation of cells was significantly (p ≤ 0.05) increased in random nanofibers compared to aligned nanofibrous scaffold (Fig 4).

**Fig 5:**
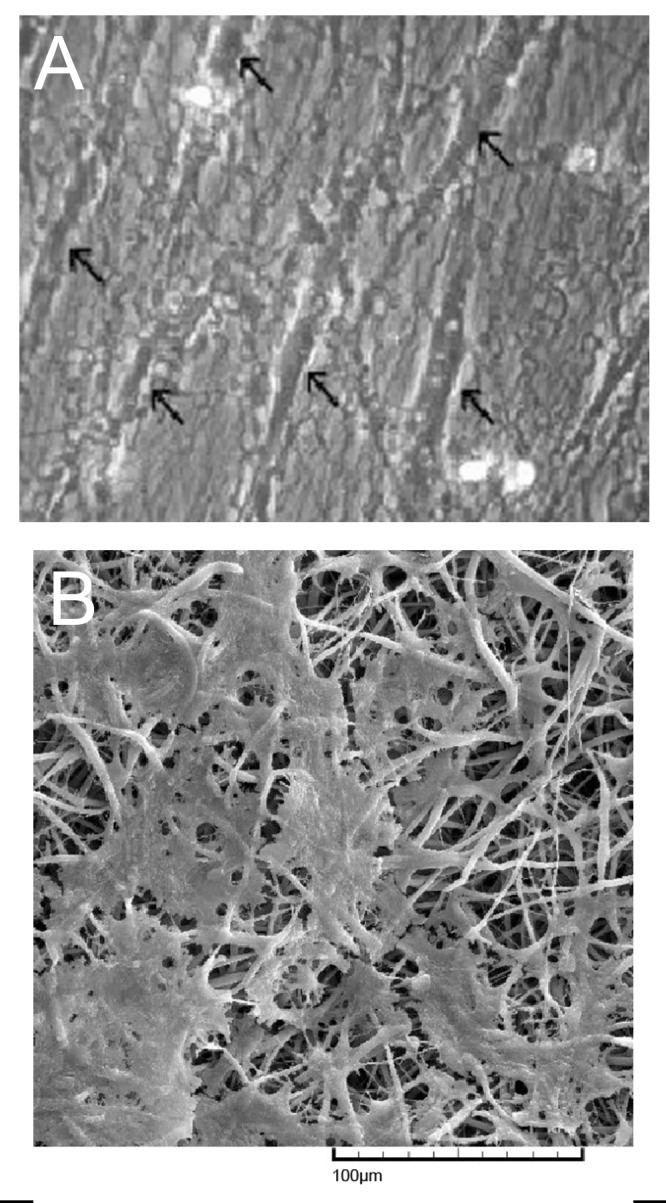
SEM micrograph of MSCs on nanofiber scaffolds obtained after 1 day of cell culture: (A) aligned nanofiber (B) random nanofiber. Arrows indicate mesenchymal cells along aligned fibers.

SEM images of cell cultures observed on day 1 (Fig 5) showed normal morphology of mesenchymal cells on p-PCL aligned and random nanofibrous scaffolds. Cells on p-PCL aligned nanofibrous scaffolds showed normal extensions and spindle-shaped morphology. Cells oriented along the direction of the fibers and clustered around the aligned fibers in a longitudinal fashion (Fig 5A) while the random fibers showed cells oriented in different directions on the nanofibers (Fig 5B).

## Discussion

The shortage of donor organs and the high cost and possible complications of transplant surgery have created the need for an alternative source of mammalian tissue. Recently, tissue engineering research has provided the macrolevel structures to form real-size organ/tissue using biomaterial and precursor cells. It is clear that adequate tissue renewal won’t be possible without its nanostructure reconstruction. Electrospun nanofibrous scaffolds are capable of supporting cellular attachment and proliferation during *in vitro* culture (7, 15). We discuss the concept of using a MSCs-bionanomaterial approach for tissue engineering applications. PCL has been extensively used for tissue engineering due to its high tensile strength, non-toxic nature and biodegradability, but its hydrophobic character is undesirable for *in vitro* cell culture (7, 16). Many studies have shown that scaffolds with hydrophilic surfaces show better cell adhesion and growth. In other hand, cell attachment and proliferation on the surface of biomaterials increases with the increase in surface hydrophilicity (17-20). Blending of synthetic polymer and natural polymer is one of the methods to introduce functional groups such as amine, hydroxyl and carboxyl groups to provide scaffolds with a more hydrophilic character (20). For example, poly (acrylamide) as hydrophilic polymers grafted on PCL surfaces showed improved cell adhesion (21). Plasma treatment, however, serves as an effective substitute for such modification procedures, since it uses no solvents nor generates chemical waste, and is associated with less polymer degradation. Enhancement of surface hydrophilicity can be induced by having oxygen-containing groups (–OH, – COOH etc) on the surface of polymers. In this respect, plasma treatment is a rapid, non-solvent surface modification process, which can create active sites on the surface of polymeric materials (3). Many studies have stated plasma surface treatment of scaffolds with N_2_, O_2_ and NH3 makes the polymer surface more hydrophilic, more polar and more bio-adhesive (17, 18).

 O_2_ plasma treatment is a surface modification selective process that influences the surface wettability of polymeric scaffolds. The present study attempted to improve the efficacy of PCL nanofibers by a short duration plasma treatment process to enhance the bioactivity of the scaffolds and provide an ideal substrate for tissue engineering applications. In the present work, water drops penetrated rapidly into the PCL nanofibrous scaffolds after plasma treatment. The surface contact angle of p-PCL scaffolds was lower than that of PCL scaffolds, showing an improved hydrophilicity compared to PCL scaffolds. Moreover, it was found that the hydrophilicity of p-PCL decreased its tensile stress.

Similar results were observed in work by Prabhakaran et al. who utilized p-PCL nanofibers to enhance the biological effects of PCL and improve cell growth and proliferation. In common with our findings, Prabhakaran et al. also showed that the water contact angle of nanofiber was lower compared to that on PCL scaffolds. They also showed that the number of MSCs attached to p-PCL scaffolds was not only higher than the number attached to PCL scaffolds but also higher than the number of MSCs attached on PCL/collagen scaffolds (3). In addition, Siri et al. showed that PCL scaffold treated with air plasma was less hydrophobic and exhibited a 66% increase in cell adherence compared to the original scaffold (22). Reports by Shabani et al. showed enhancement in hydrophilicity after plasma treatment (23). However, our studies did not involve comparative proliferation efficacy testing. Therefore, we selected plasma treated PCL for cell culture study due to its suitable hydrophilic properties.

Both the SEM and MTT assay showed that MSCs can attach and grow on p-PCL scaffolds. Also p-PCL nanofibrous scaffolds improve cell adhesion and proliferation compared with TCP. Our results are similar to those reported by Srouji et al. for human MSC culture on PCL/Collagen nanofiber (15). The results of our study also showed that the proliferation of MSCs was increased on random nanofibers compared to aligned fibers, a possible reason for this being that random fibers contain lots of interconnected pores and rough surfaces that assist adhesion and proliferation of a greater number of cells. Similar results have been reported by Deepika Gupta for the culture of MSCs on random PCL scaffolds.

The results of our study showed that electrospinning provides a nanoscale environment for cell arrangement to further remodel the ECM, and the surface modification of PCL nanofibrous scaffolds by plasma treatment serves as an effective and convenient method for improving the hydrophobic characterization of nanofiber. Futhermore, cell viability and cell morphology indicated that the electrospun treated PCL scaffold better supported growth of the rat bone marrow derived MSCs compared to the 2-D TCP culture system.

## Conclusion

The major challenge in tissue engineering is to design and fabricate a biodegradable scaffold with a surface suited to cell attachment, proliferation and differentiation, and one that can assist in the process of tissue formation. Evaluating the *in vitro* biocompatibility of plasma treated PCL nanofibrous scaffolds using MSCs showed comparatively higher MSC proliferation on p-PCL nanofibrous scaffolds than TCP. Results of our study suggested that although aligned nanofiber scaffolds are useful in tissue engineering, random nanofiber scaffolds better supported the cell proliferation process. These findings highlight the potential of simple plasma treatment to transform PCL nanofibrous scaffolds into ideal substrates for tissue engineering applications.
